# Lexical feature analysis of Chinese informed consent forms based on the information entropy methods: A paired study of minor and their guardian’ version

**DOI:** 10.1371/journal.pone.0338611

**Published:** 2025-12-15

**Authors:** Qiansu Yang, Yining Wang, Wenbin Shi, Zhenzhen Li, Hong Liang, Jiang Cao, Nan Bai, Chien-Hung Yeh

**Affiliations:** 1 School of Information and Electronics, Beijing Institute of Technology, Beijing, China; 2 Department of Pharmacy, Medical Supplies Center of Chinese PLA General Hospital, Beijing, China; 3 The Key Laboratory of Brain Health Intelligent Evaluation and Intervention, Ministry of Education, Beijing, China; 4 The JiaXing Key Laboratory of Intelligent Management for CPCR and Severe Infections (A), Jiaxing, China; 5 Department of Medical Engineering, Medical Supplies Center of Chinese PLA General Hospital, Beijing, China; Roma Tre University: Universita degli Studi Roma Tre, ITALY

## Abstract

High-quality informed consent forms (ICFs) are crucial to facilitate effective communication between researchers and patients. However, the complex and specialized terminologies in ICFs often result in biased or late interpretations, thus hindering the ability to make decisions in line with their own will. Therefore, evaluating the readability of ICFs is an important task for Institutional Review Boards (IRBs) and regulatory agencies. This study proposes the use of information theory methods, including Shannon entropy and its extension—Rényi entropy (*α* values = 0, 0.5, and 1.5), as a set of comparisons, to quantify the lexical characteristics of Chinese ICFs, for either minors or their guardians. The Shannon entropy and Rényi entropy values of minor-version ICFs were significantly lower than those of guardian-version ICFs. The Shannon entropy and Rényi entropy with *α* = 1.5 of the ICFs for minors show no significant differences compared to those of the sixth-grade textbooks, while the Rényi entropy with both *α* = 0 and 0.5 shows no significant differences compared to the ninth-grade textbooks. This study utilized information entropies to assess lexical features of ICFs, as a pilot study to validate the feasibility of implementing Shannon and Rényi entropies to evaluate readability in Chinese ICFs.

## 1 Introduction

Informed consent is a critical component of clinical research [1]. It is a communication process that provides either patients or their guardians (*e.g.*, parents) with the required information on treatment and diagnosis, enabling them to make informed decisions [2]. As a fundamental prerequisite for clinical research, informed consent has been gradually incorporated into the laws of most countries over the past six decades [3], and is subject to oversight by administrations and the general public.

Informed consent comprises two main processes, *i.e.*, “informed” and “consent”. “Informed” serves as the prerequisite for “consent,” meaning that researchers must fully disclose the nature of the research, and the participant’s involvement to potential participants before their involvement [4]. Participants must be voluntary and decide whether or not to participate in the research based on a thorough understanding of the information provided [5].

The informed consent forms (ICFs), as a carrier of informed consent information, are generally regarded as a legally binding document. ICFs contain all the information that needs to be disclosed to participants, including the research background, procedures, risks, potential benefits, and compensation, among other details. High-quality ICFs are critical for ensuring effective communication of information between researchers or doctors with patients [6]. However, it is often challenging to fully and clearly explain research information to participants without medical expertise. ICFs that are filled with complex and obscure professional terminology can be difficult to understand, even for individuals with higher education or certain medical expertise. In such cases, the participants’ full understanding cannot be guaranteed, making it even harder to ensure that they make choices that are consistent with their own decisions. This also makes the function of ICFs fall into a dilemma, that is, ICFs have typically emphasized the provision of information over support to people making a difficult decision [7]. Paasche-Orlow et al. found that Institutional Review Boards (IRBs) commonly provide text for informed-consent forms that fall short of their own readability standards [8]. The mean Flesch–Kincaid scores for the readability of sample text provided by IRBs exceeded the stated standard by 2.8 grade levels. Another research also found that mind wandering increases linearly with text difficulty [9]. As reading difficulty increases and interest decreases, people are more prone to mind-wandering, which explains why more difficult texts lead to a decline in reading comprehension. Therefore, evaluating the readability of ICFs is an important task for IRBs and regulatory administrations.

In Civil Code of the People’s Republic of China [10], the civil capacity of minors is divided into three stages: (1) Minors (children) under the age of 8 are considered to have no capacity for performing civil juristic acts, and may perform a civil juristic act only through their legal representatives; (2) Minors aged 8 or above are considered to have limited civil capacity, and their civil legal acts must either be performed by their legal guardians or approved and ratified by them; (3) natural people aged 18 or above, as well as minors aged 16 or above who main source of support is the income from their own labor, are deemed as a person with full capacity for performing civil juristic acts. From the perspective of Chinese law, parents or guardians can decide on behalf of most minors whether to participate in clinical research. However, ethical principles require that these minors, as participants in clinical research, understand the research information and agree to participate as far as possible [11,12].

As the right to be informed of Chinese minors gradually gains more attention, an increasing number of clinical researchers involving minors have started to design ICFs specifically for minors to read and sign. Informing minor participants not only safeguards their right to be informed but also helps them understand the research procedures, precautions, and other details, which can be to the benefit of the research progress. However, ICFs that are difficult for even adults to read pose an even greater challenge for minors, who may lack sufficient reading ability and knowledge. This makes it impractical to include all research information in the informed consent process for minors. Therefore, IRBs and researchers need to pay more attention to whether minors can comprehend the content of the ICFs. Thus, there is an urgent need to explore a readability evaluation method for Chinese ICFs that can be broadly applied across all age groups.

Currently, there are multiple methods internationally available to evaluate the readability of medical information provided to patients, such as the Flesch-Kincaid scale [13–15], the Gunning Fog Index [16], and the Simple Measure of Gobbledygook (SMOG) test [17]. However, these readability evaluation methods are designed for English-based texts, particularly using the number of syllables as the standard for distinguishing complex words. Thus, these methods cannot be directly applied to Chinese texts. In a study on the readability of Chinese text, Yong et al. found that character frequency and lexical richness were key factors in distinguishing readability levels [18]. The higher the value of lexical richness in a text, the greater the uncertainty of the words used (indicating a higher variation in vocabulary), and consequently, greater reading difficulty for the text [19]. Shannon entropy was confirmed to be an effective measure for evaluating these indices that influence the readability of Chinese text [20–22]. In addition, aside from the inapplicability of these methods due to language differences, ICFs themselves often contain a large number of incomplete sentence structures, such as lists, phrases, and fragmentary descriptions (inclusion and exclusion criteria, et al). This makes it difficult to accurately reflect the readability level of ICFs.

The information entropy method has been widely applied in biomedical signal processing [23,24] and financial time series analysis [25]. Therefore, this research proposes the use of information theory methods, including Shannon entropy and its extension—Rényi entropy, to analyze the lexical features of Chinese ICFs for minors and their guardians. The reliability of the evaluation method will be validated by referencing Chinese language textbooks from different grade levels. This approach aims to explore the feasibility of establishing a readability evaluation system for ICFs.

## 2 Materials and methods

### 2.1 Materials

We selected the clinical research involving minors in China between January 2018 and December 2024, including investigator-initiated clinical research and drug clinical trials. We collected a total of 17 pairs of minor-version ICFs and corresponding guardian-version ICFs from 17 approved ethical review applications. All the ICF texts are written in Simplified Chinese. Since this research focuses on the textual characteristics of ICFs, we retained only the ICFs for teenagers (ages 12−18) and their guardians, and excluded those designed for children under 12 years old, as those ICFs often convey research information through visual elements such as illustrations. This study only collects and analyzes the text content of ICFs and does not contain any personal information of the human participants. The Institutional Review Board of the Chinese PLA General Hospital has granted an exemption from ethical review for this study (S2025-378–01).

As a comparison, we selected Chinese language textbooks (excluding poetry and prose of classical Chinese) from the second-grade (n = 45), sixth-grade (n = 36), and ninth-grade (n = 23) editions published by the People’s Education Press (organized and prepared by the Ministry of Education in China). The second-grade texts correspond to the reading ability of minors around 8 years old, marking the transition from no civil capacity to limited civil capacity. The sixth-grade texts correspond to the reading ability of minors around 12 years old, the age boundary for entering adolescence. The ninth-grade texts correspond to the reading ability of minors aged 15–16, marking the completion of compulsory education or the full civil capacity under certain conditions.

### 2.2 Information entropy

Shannon entropy is a core concept in information theory, put forward by Claude Shannon in 1948 to quantify the uncertainty of information [26]. It essentially measures the uncertainty or amount of information associated with the possible values of a random variable. The formula for Shannon entropy with base 2 is as follows:


$$H(P)=-\sum_{i=\text{1}}^{n}p_{i}{\text{log}}_{\text{2}}p_{i}$$

where *p*_*i*_ denotes the probability of the *i*-th possible event occurring in the given probability distribution P, while *n* represents the total number of possible outcomes in the system. The higher the Shannon entropy value, the more evenly distributed the probability density of information, indicating greater randomness in the distribution [27]. In this research, Shannon entropy is used as a standard method to evaluate text complexity and readability. A higher entropy value indicates a larger vocabulary and a more uniform word frequency distribution within the text, meaning that the text is more complex and shares less readability.

Rényi entropy, as an extension of Shannon entropy, introduces a parameter *α* to represent different weighting sensitivities. For a discrete probability distribution *P* = (*p₁*, *p₂,*..., *pn*), the Rényi entropy of order *α* (*α *> 0, *α* ≠ 1) is defined as:


$$H_{R}(P)=-\frac{\text{1}}{\alpha -\text{1}}{\text{log}}_{\text{2}}\sum_{i=\text{1}}^{n}p_{i}^{\alpha }\;\;(\alpha >\text{0}\;\text{and}\;\alpha \neq \text{1})$$

When *α* → 1, Rényi entropy converges to Shannon entropy. When *α* < 1, Rényi entropy places more emphasis on the size of the sample set while reducing the influence of the probability distribution [28]. Conversely, when *α *> 1, Rényi entropy focuses more on events with higher probabilities.

When *α* = 0, Rényi entropy is equivalent to Hartley entropy, and the formula is as follows:


$$H_{\text{0}}={\text{log}}_{\text{2}}n$$

When *α* = 0, Rényi entropy is used to measure the information content of a set of equally probable events. When analyzing text, it no longer considers the probability distribution of the words but only focuses on which words are possible. In this research, Rényi entropy represents the size of the word set, *i.e.*, the total vocabulary of the text.

When *α*→∞, Rényi entropy represents the negative logarithm of the most probable event and is commonly referred to as maximum entropy or minimum uncertainty entropy. The formula is as follows:


$$H_{\infty }(P)=-{\text{log}}_{\text{2}}(p_{max})$$

When *α*→∞, Rényi entropy becomes dominated by the maximum probability *p*_max_, which significantly influences the result, while other smaller probabilities rapidly approach 0. At this point, Rényi entropy no longer considers the entire probability distribution but focuses solely on the maximum probability value. When analyzing text, Rényi entropy represents the proportion of the most frequently occurring word, reflecting the distribution of the most dominant informational feature in the text.

Therefore, in this research, to simultaneously focus on the size of the text word set and its probability distribution, we chose Rényi entropy across *α* ∈ [0,2] with 0.1 steps (replaced by Shannon entropy when *α* = 1) to analyze the ICFs.

### 2.3 Data preprocessing

In this research, information such as the cover page, signature page, headers, and footers of the ICFs was removed, while the text within tables and text boxes was retained, and the text format was standardized. For the comparison of Chinese language textbooks, images, introductory sections, and footnotes within the textbooks were removed, and the text format was similarly standardized.

We used the open-source toolkit (jieba: https://github.com/randoruf/jieba-chinese-tokenization) to perform word segmentation on the ICFs. After segmentation, punctuation marks, spaces, and other characters were removed from the segmentation results, retaining only the lexical information.

### 2.4 Statistics

Continuous variables are represented by means ± standard deviation (SD) or median (lower quartile, upper quartile). The Wilcoxon signed-rank test was used to assess the median difference of Shannon entropy and Rényi entropy between ICFs for minors and their guardians. The area under the curve (AUC), based on logistic regression, was calculated to discriminate between minor-version and guardian-version ICFs using Shannon entropy or Rényi entropy. Furthermore, the Shannon entropy and Rényi entropy of ICFs designed for minors were compared with those of textbooks for second, sixth, and ninth graders. A *P*-value of less than 0.05 is considered significant.

## 3. Results

### 3.1 Method verification

**Fig 1:**
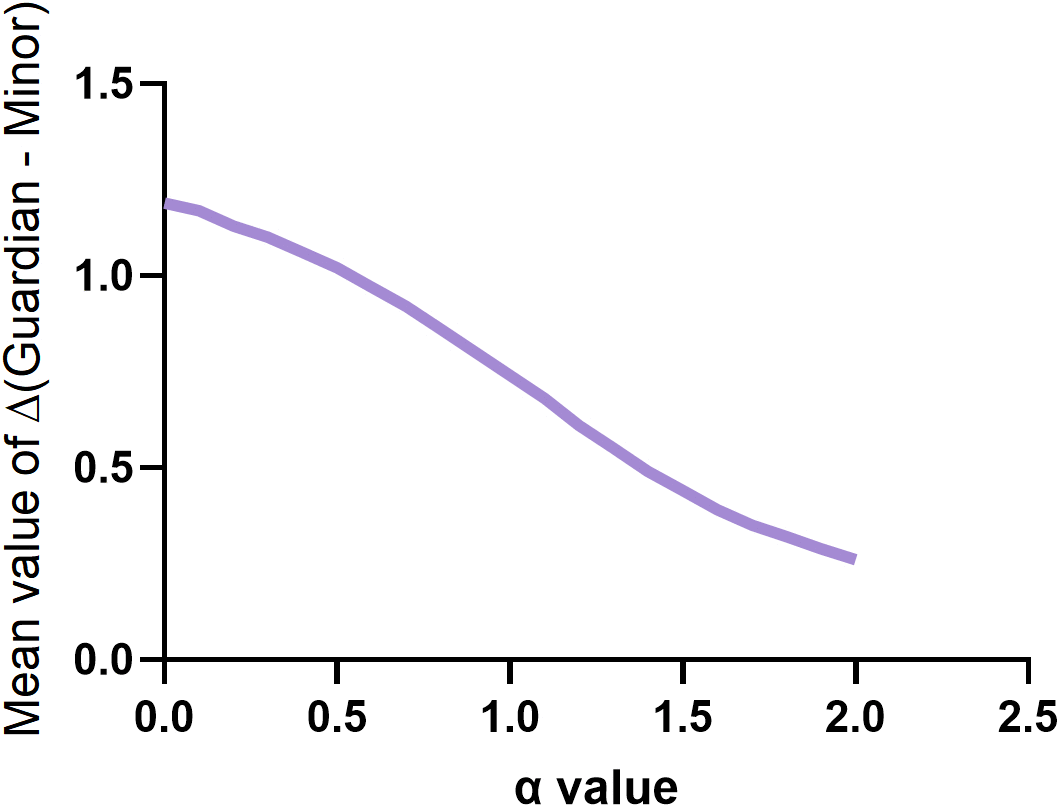
Parameter sensitivity analysis of entropy differences between Guardian and Minor ICFs across α values.

The minor-version ICFs and guardian-version ICFs were used to calculate Rényi entropy across *α*∈[0, 2] with steps of 0.1 (replaced by Shannon entropy when *α *= 1). The paired differences ∆(Guardian – Minor) were then computed, and the corresponding curves were plotted (Table 1 and 2). The results showed that ∆ decreased as *α* increased, with no distinct separation peaks observed in the overall curve (Fig 1). A random experiment (Section 3.6) indicated that above *α* = 1.6, the minor-version ICFs and guardian-version ICFs no longer exhibited significant differences (*P* > 0.05). Therefore, in the subsequent analyses, we reduced the step size of *α*, selecting 0, 0.5, 1 (replaced by Shannon entropy), and 1.5 for lexical feature analysis. The complete results for *α* ∈ [0, 2] are provided in the supplementary materials.

**Table 1:** Descriptive results of minor-version ICFs, guardian-version ICFs, and Chinese language textbooks of Grades 2, 6, and 9.

		Minor Ver. ICFs	Guardian Ver. ICFs	Text-Grade 2	Text-Grade 6	Text-Grade 9
Count		17	17	45	36	23
Word Count	25%	1160.50	3803.50	133.00	416.50	846.00
	Median	1714.00	5993.00	176.00	595.00	1208.00
	75%	2789.00	7210.50	238.00	791.25	1592.00
Rényi Entropy (*α* = 0)	25%	8.67	9.90	6.37	7.95	8.58
	Median	9.03	10.27	6.75	8.32	9.12
	75%	9.72	10.43	7.06	8.84	9.44
Rényi Entropy (*α* = 0.5)	25%	8.15	9.30	6.20	7.72	8.31
	Median	8.38	9.45	6.58	8.07	8.75
	75%	9.10	9.63	6.88	8.56	9.10
Shannon Entropy	25%	7.38	8.23	6.01	7.30	7.90
	Median	7.71	8.34	6.37	7.70	8.20
	75%	8.13	8.57	6.60	7.98	8.55
Rényi Entropy (*α* = 1.5)	25%	6.47	7.02	5.74	6.87	7.19
	Median	7.04	7.17	6.10	7.12	7.50
	75%	7.27	7.49	6.31	7.36	7.69

**Table 2:** The Wilcoxon signed-rank test results of Shannon entropy and Rényi entropy (*α* values: 0, 0.5, and 1.5) between ICFs for minors and their guardians.

	*P*-value	Positive Difference	Negative Difference
Rényi Entropy (*α* = 0)	< 0.001	17	0
Rényi Entropy (*α* = 0.5)	< 0.001	16	1
Shannon Entropy	0.001	14	3
Rényi Entropy (*α* = 1.5)	0.025	10	7

**Fig 2:**
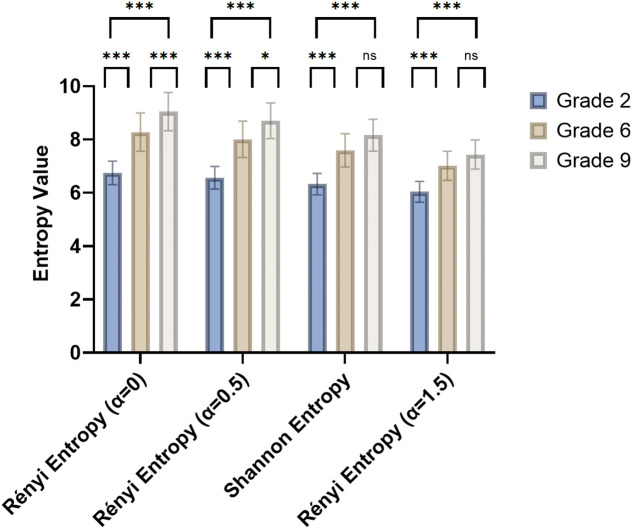
The entropy values of the Chinese language textbooks for the second, sixth, and ninth grades.

Shannon entropy and Rényi entropy (*α* values of 0, 0.5, and 1.5) were chosen to analyze the Chinese language textbooks for the second, sixth, and ninth grades (Fig 2). The results showed that as the difficulty of the texts increased, their entropy values also increased. This preliminarily proves that Shannon and Rényi entropy can be used for text readability assessment.

**Fig 3:**
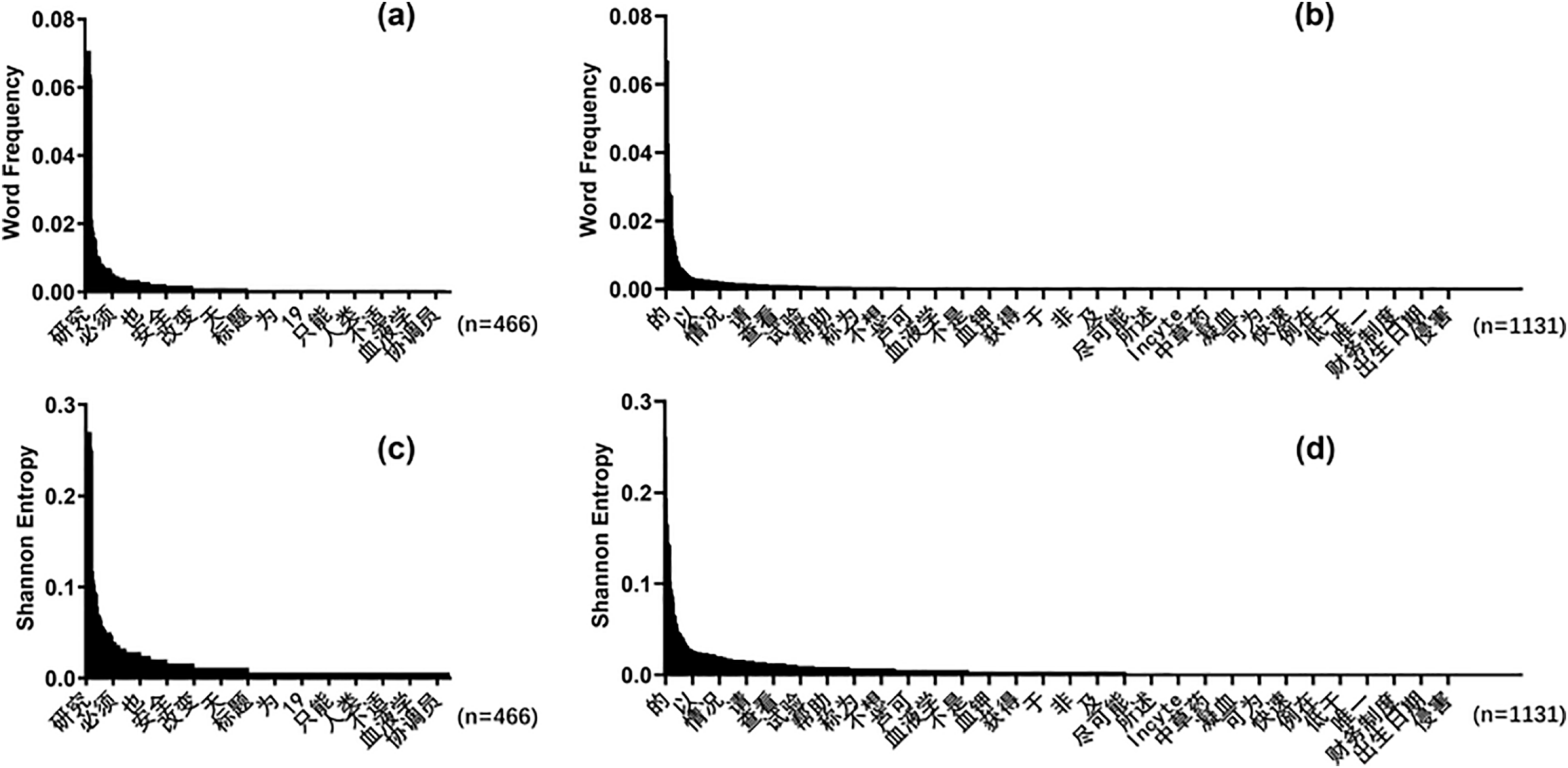
Word frequency and shannon entropy distribution. This figure shows the distribution of word frequency (a. Minor-version; b. Guardian-version) and Shannon entropy (c. Minor-version; d. Guardian-version) for one set of minors and their guardians ICF.

We chose one set of minors’ and their guardians’ ICF to analyze the distribution of the word frequency and Shannon entropy for each single word. The results shown in Fig 3 (a) and (b) indicate that compared with the guardian-version ICF, the word frequency distribution of the minor-version ICF is more uneven. Fig 3 (c) and (d) display the contributions of Shannon entropy for single words in ICF. Similarly, the distribution of Shannon entropy contributions of the ICF for minors is more uneven than that of the ICF for guardians. Since Rényi entropy is based on the overall distribution of word frequencies in the entire text, it can not calculate the contribution of a single word to the Rényi entropy value.

### 3.2 Descriptive statistics

The descriptive results, including word count, Shannon entropy, and Rényi entropy (*α* values: 0, 0.5, and 1.5), of minor-version ICFs, guardian-version ICFs, and Chinese language textbooks from the second grade, sixth grade, and ninth grade were shown in Table 1.

### 3.3 Comparisons of minor-version with guardian-version ICFs

**Fig 4:**
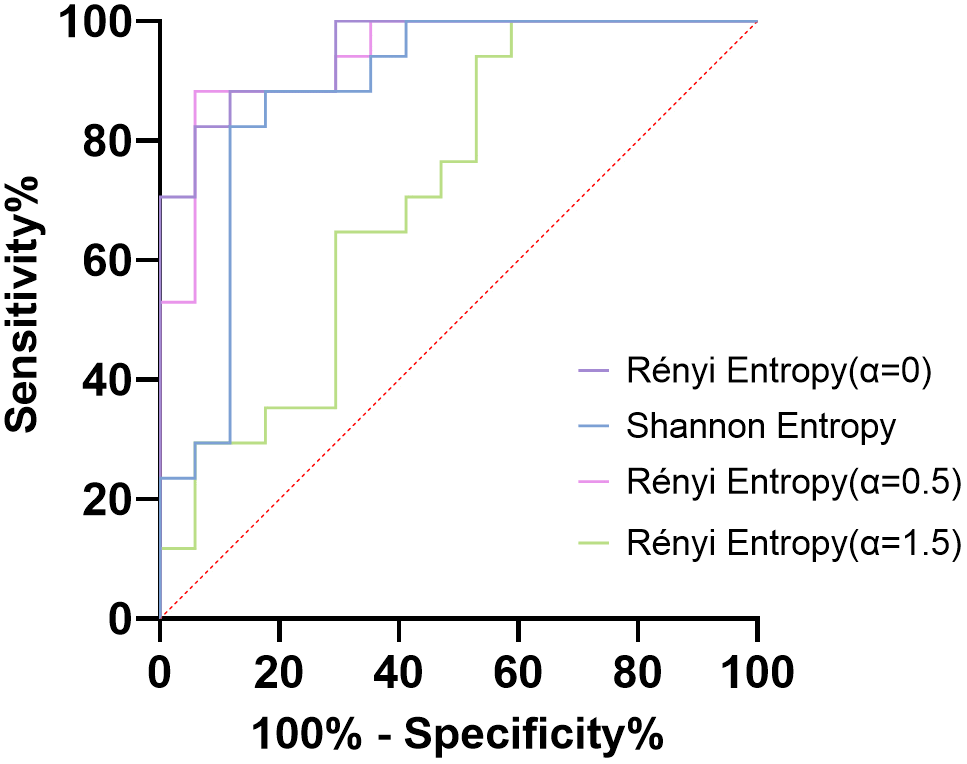
The receiver operating characteristic (ROC) curves for the discrimination between minor-version and guardian-version ICFs. The performance of four different entropy measures (Shannon entropy and Rényi entropy with α-values of 0, 0.5, and 1.5) was shown.

The Wilcoxon signed-rank test results of Shannon entropy and Rényi entropy (*α* values: 0, 0.5, and 1.5) between ICFs for minors and their guardians are shown in Table 2. The Shannon entropy of minor-version ICFs was significantly lower than that of guardian-version ICFs (7.71 (7.38, 8.13) vs. 8.34 (8.23, 8.57), *P* = 0.001). Significant differences were also found in the Rényi entropy (*α* = 0) (9.03 (8.67, 9.72) vs. 10.27 (9.90, 10.43), P < 0.001), Rényi entropy (*α* = 0.5) (8.38 (8.15, 9.10) vs. 9.45 (9.30, 9.63), *P* < 0.001) and Rényi entropy (*α* = 1.5) (7.04 (6.47, 7.27) vs. 7.17 (7.02, 7.49), *P* = 0.025) of minors’ and their guardians’ ICFs. In distinguishing the minor-version ICFs from guardian-version ICFs, the Rényi entropy (*α* = 0) had the best performance (AUC = 0.952), followed by Rényi entropy (*α* = 0.5) (AUC = 0.941), Shannon entropy (AUC = 0.879), and Rényi entropy (*α* = 1.5) (AUC = 0.713) (Fig 4). The AUC values of four kinds of entropy and the corresponding cutoff points are shown in Table 3.

**Table 3:** The AUC values of four different entropy measures (Shannon entropy and Rényi entropy with *α*-values of 0, 0.5, and 1.5) and their corresponding cutoff points.

	AUC	Threshold	Sensitivity	Specificity
Rényi Entropy (*α* = 0)	0.952	9.78	0.882	0.882
Rényi Entropy (*α* = 0.5)	0.941	9.24	0.882	0.941
Shannon Entropy	0.879	8.22	0.824	0.882
Rényi Entropy (*α* = 1.5)	0.713	6.77	0.941	0.471

### 3.4 Comparisons of minor-version ICFs with Chinese language textbooks

**Fig 5:**
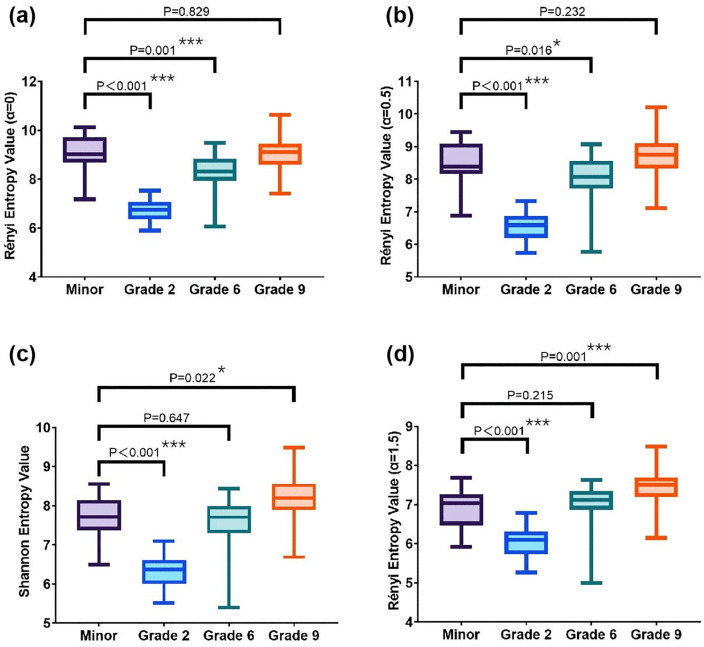
Entropy comparison across minor-version ICFs and textbooks. This figure presents the comparison of (a) Rényi entropy (α = 0), (b) Rényi entropy (α = 0.5), (c) Shannon entropy, and (d) Rényi entropy (α = 1.5), between minor-version ICFs and textbooks of different grades.

The Shannon entropy and Rényi entropy (*α*-values: 0, 0.5, and 1.5) of minor-version ICFs were significantly higher than those of Chinese language textbooks from the second grade (all *P* < 0.001). When contrasted with six-grade textbooks, minors’ ICFs showed significantly elevated Rényi entropy at *α* = 0 (*P* = 0.001) and *α* = 0.5 (*P* = 0.016), but comparable Shannon entropy and Rényi entropy (*α* = 1.5). On the contrary, no significant differences were found in the Rényi entropy (*α* = 0, 0.5) between ICFs for minors and ninth-grade textbooks. The Shannon entropy (*P* = 0.022) and Rényi entropy (*α* = 1.5) (*P* = 0.001) of minor-version ICFs were significantly lower than that of ninth-grade textbooks. These comparisons in textual complexities are shown in Fig 5.

### 3.5 The effects of textual content upon entropy

To investigate the reasons underlying the changes in information entropy between Minor and Guardian version ICFs, we divided the informed consent forms into four sections: introduction, procedures, risks and benefits, and participants’ rights and other information. We then recalculated the entropy for each section, computed the difference ∆(Guardian – Minor), and visualized the results using a heatmap (Fig 2 and Table 3). The findings indicate that the section “participants’ rights and other information” exhibits the largest variation with respect to *α*. Specifically, the results show that the sample size of Minor ICFs differs substantially from that of Guardian ICFs when *α* = 0, whereas the word frequency distribution becomes more uniform when *α* = 1.5 (Table 4).

**Table 4:** The entropy value of the difference ∆(Guardian – Minor) for each section.

	Introduction	Procedures	Risk and benefit	Participants’ rights and other information
Rényi Entropy (*α* = 0)	0.93	1.25	1.71	1.26
Rényi Entropy (*α* = 0.5)	0.84	1.10	1.55	1.07
Shannon Entropy	0.69	0.89	1.31	0.75
Rényi Entropy (*α* = 1.5)	0.50	0.70	1.00	0.39

### 3.6 Random experiment

Entropy is sensitive to vocabulary growth. Therefore, we designed a random experiment to test whether the smaller entropy values in minors’ ICFs were simply due to their shorter length compared with the guardian version. We performed additional analyses by randomly sampling 1,000 tokens from each ICF (with replacement for shorter texts) and computed the entropies over 100 iterations, then averaged the results. The results show that minor-version ICFs still exhibited significantly lower entropy values (*P* < 0.05) than guardian-version ICFs when *α* ≤ 1.5 (Table 5). Therefore, it is reasonable that this study chose Shannon entropy and Rényi entropy with *α* = 0, 0.5, and 1.5.

**Table 5:** The Wilcoxon signed-rank test results of a Random experiment.

	*P*-value	Positive Difference	Negative Difference
Rényi Entropy (*α* = 0)	0.002	15	2
Rényi Entropy (*α* = 0.1)	0.002	15	2
Rényi Entropy (*α* = 0.2)	0.002	14	3
Rényi Entropy (*α* = 0.3)	0.004	13	4
Rényi Entropy (*α* = 0.4)	0.004	13	4
Rényi Entropy (*α* = 0.5)	0.006	12	5
Rényi Entropy (*α* = 0.6)	0.006	12	5
Rényi Entropy (*α* = 0.7)	0.007	12	5
Rényi Entropy (*α* = 0.8)	0.010	11	6
Rényi Entropy (*α* = 0.9)	0.011	11	6
Shannon Entropy	0.015	11	6
Rényi Entropy (*α* = 1.1)	0.025	11	6
Rényi Entropy (*α* = 1.2)	0.031	10	7
Rényi Entropy (*α* = 1.3)	0.035	10	7
Rényi Entropy (*α* = 1.4)	0.039	10	7
Rényi Entropy (*α* = 1.5)	0.044	10	7
Rényi Entropy (*α* = 1.6)	0.055	10	7
Rényi Entropy (*α* = 1.7)	0.068	10	7
Rényi Entropy (*α* = 1.8)	0.084	10	7
Rényi Entropy (*α* = 1.9)	0.113	10	7
Rényi Entropy (*α* = 2.0)	0.136	10	7

## 4 Discussion

This study conducted a comparative analysis of the differences in Shannon entropy and Rényi entropy between minor-version and guardian-version ICFs, as well as between minor-version ICFs and Chinese textbooks from various grade levels. Significant differences were observed between the ICFs for minors and their guardians across all four entropy values. The result also showed that the readability level of the minor-version ICFs corresponds to grades six to nine, which matches the reading ability of minors aged 12–16. However, in terms of length, the word count of the minors’ ICFs far exceeds that of ninth-grade textbooks. These results are of significant importance in guiding the drafting of ICFs and evaluating their readability.

### 4.1 Differences in word set size and word frequency between minor-version and guardian-version ICFs

In this study, significant differences were observed between the minor-version and guardian-version ICFs across all four entropy values, indicating differences in word set size and word frequency between the two versions. The Rényi entropy (*α* = 1.5) shows that the probability distribution in the minor-version ICFs is less uniform than that in the guardian-version ICFs, meaning that high-frequency words (such as function words) dominate, and the information is more concentrated. On the other hand, this could also indicate simpler or repetitive sentence structures and lower lexical diversity. However, a notable number of minors’ ICFs (7/17) had Rényi entropy values (*α* = 1.5) higher than the guardians’ ICFs. We believe that the minor-version ICFs have reduced length in sections such as Risk, Benefit, and Participants’ Rights, compared to the guardian-version ICFs. This reduction led to a higher occurrence of low-frequency terms, such as technical or medical terminology, in the minor ICFs, resulting in a higher Rényi entropy (*α* = 1.5). The results in section 3.5 also show that the gap in entropy values between the Risk and Benefit and Participants’ Rights and Other Information sections rapidly reduces as the *α* value increases, thus supporting this hypothesis.

To further explore this hypothesis, we initially built a shortlist of 563 technical terms. The technical-term density (TT) was calculated as follows:


$$TT=\frac{number\;of\;technical\;terms}{total\;number\;of\;words}$$

Subsequent analysis was conducted on the TT results to examine whether the Rényi entropy parameter (*α* = 1.5) increases with higher technical-term density, using Spearman’s rank correlation coefficient (*ρ*) across ICFs. The results reveal a significant correlation between the Rényi entropy value (*α* = 1.5) and the TT results (*ρ* = 0.570, *P* < 0.001).

### 4.2 The writing of informed consent forms should aim to lower the threshold for understanding the information

Although according to Cognitive Development Theory [29,30], minors aged 12 and above are beginning to develop abstract thinking abilities, these abilities are still in the developmental stage. Complex sentence structures and medical terminology remain significant challenges for minors’ comprehension of abstract concepts. This finding highlights the need for researchers and IRBs to focus on the use of complex vocabulary and sentence structures when drafting and reviewing ICFs, rather than simply reducing content by cutting text from the ICFs for guardians. In addition, the guardian-version ICFs significantly surpass the reading ability typically acquired by individuals upon completing compulsory education. Therefore, in most cases, participants and their guardians are required to make decisions that exceed the limits of their available information and cognitive abilities. This also determines that the drafting of ICFs is an extremely important work, which needs to lower the threshold of information understanding as much as possible, so that participants can fully understand the research information.

The Shannon entropy and Rényi entropy (*α* = 1.5) of the minor-version ICFs show no significant differences compared to sixth-grade textbooks, while the Rényi entropy with *α* = 0 and 0.5 shows no significant differences compared to ninth-grade textbooks. The entropy values of the minors’ ICFs generally align with the grades six to nine, but the length far exceeds that of textbooks. According to Cognitive Load Theory [31,32], human working memory has a limited capacity during learning and information processing, and longer texts may increase cognitive load during reading [33]. Extended text length can significantly impact the effectiveness of reading and understanding informed consent forms by participants [34,35]. Therefore, researchers and IRBs need to consider the length of ICFs during their drafting and review. They could extract key information from the ICFs to create summaries or organize the information in layers based on its importance, so that the primary informed content is concentrated within specific sections. When reading ICFs, participants may not receive sufficient explanations. Although this research shows that the readability of the minor-version ICFs is equivalent to the reading level of grades six to nine, the reading environment of ICFs is significantly different from the learning environment of Chinese language classes. We should not overly expect researchers to read and explain ICFs line by line, as teachers would do with textbooks, especially since ICFs themselves do not come with well-established instructional methods like those for classroom texts. Similarly, designing ICFs faces a comparable challenge. Expecting researchers to craft a text that is both easy to understand and linguistically engaging, like a professional writer, is clearly unrealistic.

### 4.3 The construction of a readability evaluation tool for Chinese ICFs based on information entropy is feasible

The study demonstrates that Shannon and Rényi entropy values increase as the difficulty of the text rises, as seen in the comparison of ICFs across different versions (minor and guardian) and Chinese language textbooks of various grade levels (second, sixth, and ninth grades). The entropy values for minor-version ICFs and guardian-version ICFs, as well as textbooks, reflect the overall text complexity, which can be correlated with how easily the target audience—minors and their guardians—can comprehend the document. Shannon entropy and Rényi entropy can successfully capture the complexity and variability in the text, providing objective measures of its readability.

The random experiment is designed to test the effects of text length on entropy values showed that even when adjusting for text length by randomly sampling the minor-version ICFs still exhibited significantly lower entropy values compared to the guardian-version ICFs (*α *≤ 1.5). This strengthens the argument that the differences in entropy are not solely due to text length, but rather reflect inherent differences in text complexity between the two versions.

Furthermore, entropy can also be analyzed at different granular levels (*e.g.*, Introduction, Procedures, Risk, and Benefit), enabling more targeted interventions. This could lead to a more efficient and systematic approach to revising ICFs, ensuring that they are both legally sound and comprehensible to a wide range of participants.

### 4.4 The advantages and disadvantages of Shannon and Rényi entropy

Entropy measures are critical for evaluating complex, non-linear systems, much like how they apply entropy to electrophysiological data [36,37]. In the study of text readability and linguistic feature analysis, Shannon entropy, with its clear probabilistic interpretation and robust theoretical foundation, offers strong interpretability and empirical reliability in quantifying the average uncertainty of a text [38]. It effectively captures the “average information content” at both lexical and syntactic levels and has been validated across multiple languages and genres in readability research [39]. Because it originates directly from fundamental information theory, the computation and interpretation of Shannon entropy are relatively straightforward, making it particularly suitable for constructing general frameworks of linguistic complexity measurement. However, its main limitation lies in its fixed sensitivity: Shannon entropy assigns equal weight to high-frequency and low-frequency events, which makes it less flexible when distinguishing between the contributions of frequent and rare words to readability [40]. In contrast, the parameterized nature of Rényi entropy enables a tunable analytical framework, offering notable advantages in handling heterogeneous text structures and mitigating the effects of noisy linguistic data. However, this flexibility comes at the cost of increased interpretive and computational complexity. The selection of the parameter *α* remains largely heuristic, lacking standardized guidelines, and its interpretation is highly dependent on the specific linguistic or analytical context. Consequently, a combined application of Shannon and Rényi entropy can yield a hierarchical and conceptually coherent framework for the comprehensive analysis of Chinese ICF lexical features.

### 4.5 Limitations of the study

One limitation of the current study could be that our focus on the choice of different word segmentation tools or tokenization tools can have an impact on the results. To avoid the impact of this difference, we choose the most popular open-source word segmentation toolkit (jieba) for text segmentation, and carry out lexical feature analysis under the same conditions. Another limitation of this work may be based solely on information entropy and lacks an analysis of the relationships between words; thus, the lexical relation of Chinese text requires further exploration. For example, terminologies and their explanations are not analyzed together in terms of readability. Finally, the information conveyed by ICFs is multidimensional, including elements such as charts, images, and more. The readability of the ICFs is not only influenced by lexical information, but also by different layouts and formatting can also affect the readability. However, given that the main purpose of the present study is to discover lexical features for ICFs, exploring such issues is beyond our scope.

## 5 Conclusions

The design of ICFs is a complex engineering that involves knowledge from multiple fields, such as medicine, ethics, law, linguistics, psychology, and education. Unlike literary works, the purpose of an ICF is to ensure that participants fully understand the research procedures, risks, and benefits. Therefore, when designing ICFs, it is necessary to comprehensively consider various factors such as participants’ cultural background, educational level, and cognitive abilities to ensure that the content, structure, and language expression effectively convey the core information. For IRBs and researchers, faced with increasingly stringent review requirements, the help of digital tools to quantitatively evaluate ICFs will become a necessary approach.

This research utilized the method of information entropy to analyze the lexical features of ICFs and has preliminarily validated the feasibility of applying Shannon entropy and Rényi entropy to the readability evaluation of Chinese ICFs. With the advancement of digital technologies, the design of ICFs can also explore personalized approaches, and provide auxiliary reading functions according to different levels of reading ability to ensure a thorough understanding of the research information.
